# Effect of saccharin supplementation on weight gain, caloric intake and basal oxygen consumption in Wistar rats

**DOI:** 10.1186/1758-5996-7-S1-A158

**Published:** 2015-11-11

**Authors:** Denise Entrudo Pinto, Kelly C Foletto, Pedro Dal Lago, Ramiro Barcos, Marcello Bertoluci

**Affiliations:** 1 Universidade Federal do Rio Grande do Sul, Porto Alegre, Brazil

## Background

The use of non-caloric sweeteners can interfere in the regulation of appetite, promoting greater food intake and weight gain. In previous data, our results showed that animals who consumed yogurt with saccharin and aspartame had a increase in weight compared to the group using sucrose. However, as the total calorie intake was similar between the groups, we speculated that weight gain might be associated with decreased energy expenditure induced by artificial sweetener.

## Aim

Determine the caloric expenditure at rest in rats receiving saccharin or sucrose for 12 weeks.

## Materials and methods

We conducted a controlled experiment with adult male Wistar rats randomly divided into 3 groups: non-caloric sweetener (saccharin-SAC); calorie sweetener (sucrose-SUC) or control ( non sweetened yogurt -CON) given daily over a period of 12 weeks with free chow and water. Weight gain, food intake and water control were determined weekly, basal oxygen consumption was measured at 0, 5 and 12 weeks. We used one-way ANOVA with Dunnett's test and ANOVA by repeated measures and mixed model assessment.

## Results

The SAC group promoted greater weight gain than control (p=0.031). All groups had similar total caloric intake. The maximal oxygen consumption was not diferent between groups during the whole experiment, respectivelly: SAC (basal 27.72±1.91; 5 weeks 28.39±1.96 and 12 weeks 27.16±0.87), SUC (basal 28.66±1.96; 5 weeks 29.35±3.16 and 12 weeks 29.08±1.61) and CONT (basal 27.16±0.87; 6 weeks 28.15±2.53 and 12 weeks 27.58±0.97).

## Conclusion

The cumulative weight gain in the animals fed with saccharin can not be attributed to a reduction in energy expenditure. Further studies are necessary to determine metabolic causes for weight gain induced by saccharin in rats.

**Figure 1:**
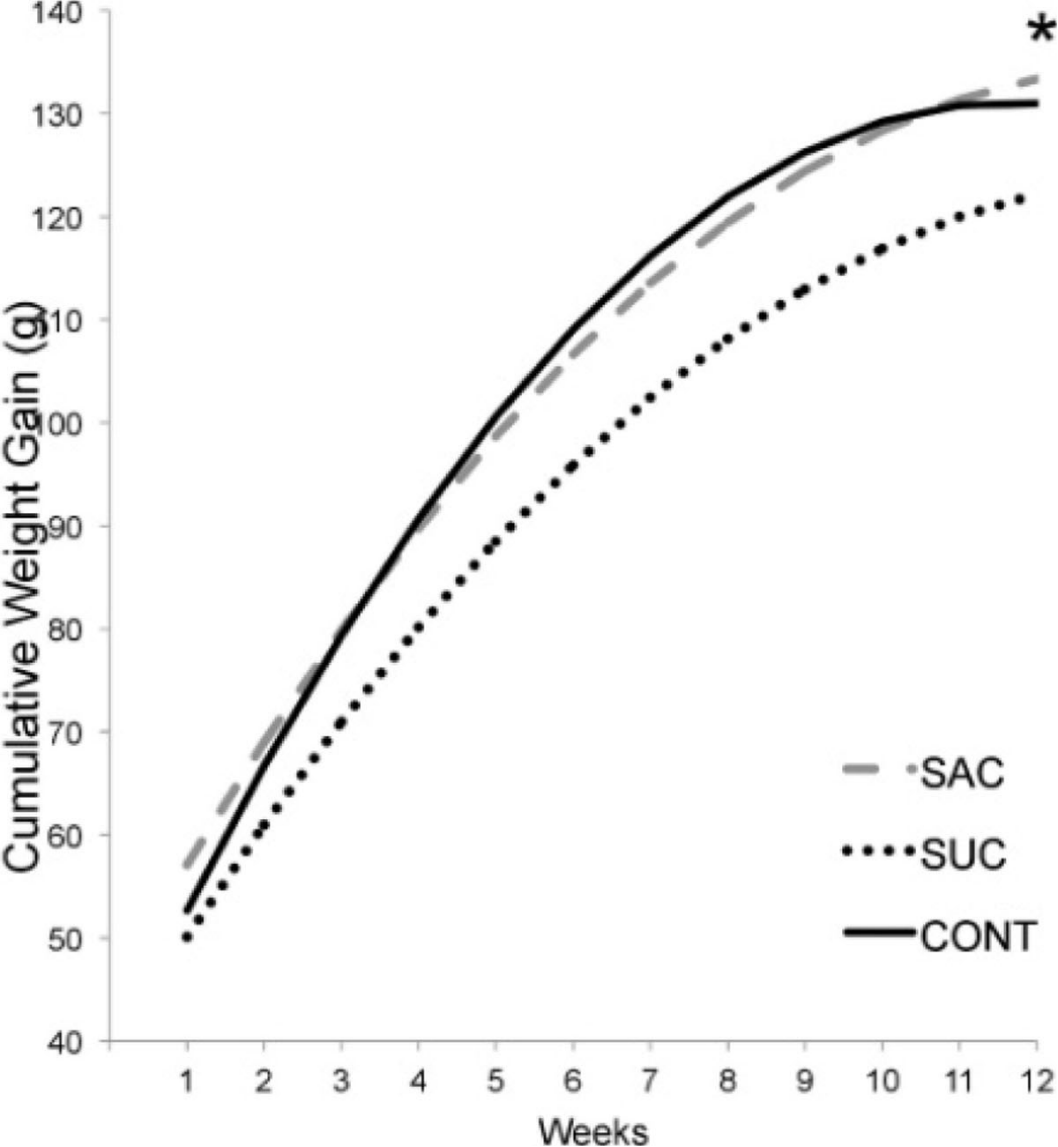
Cumulative weight gain (g) over the 12 weeks. Asterisks indicate comparisons in relations to SAC. * p <0.05 SAC in relations to CONT.

**Figure 2:**
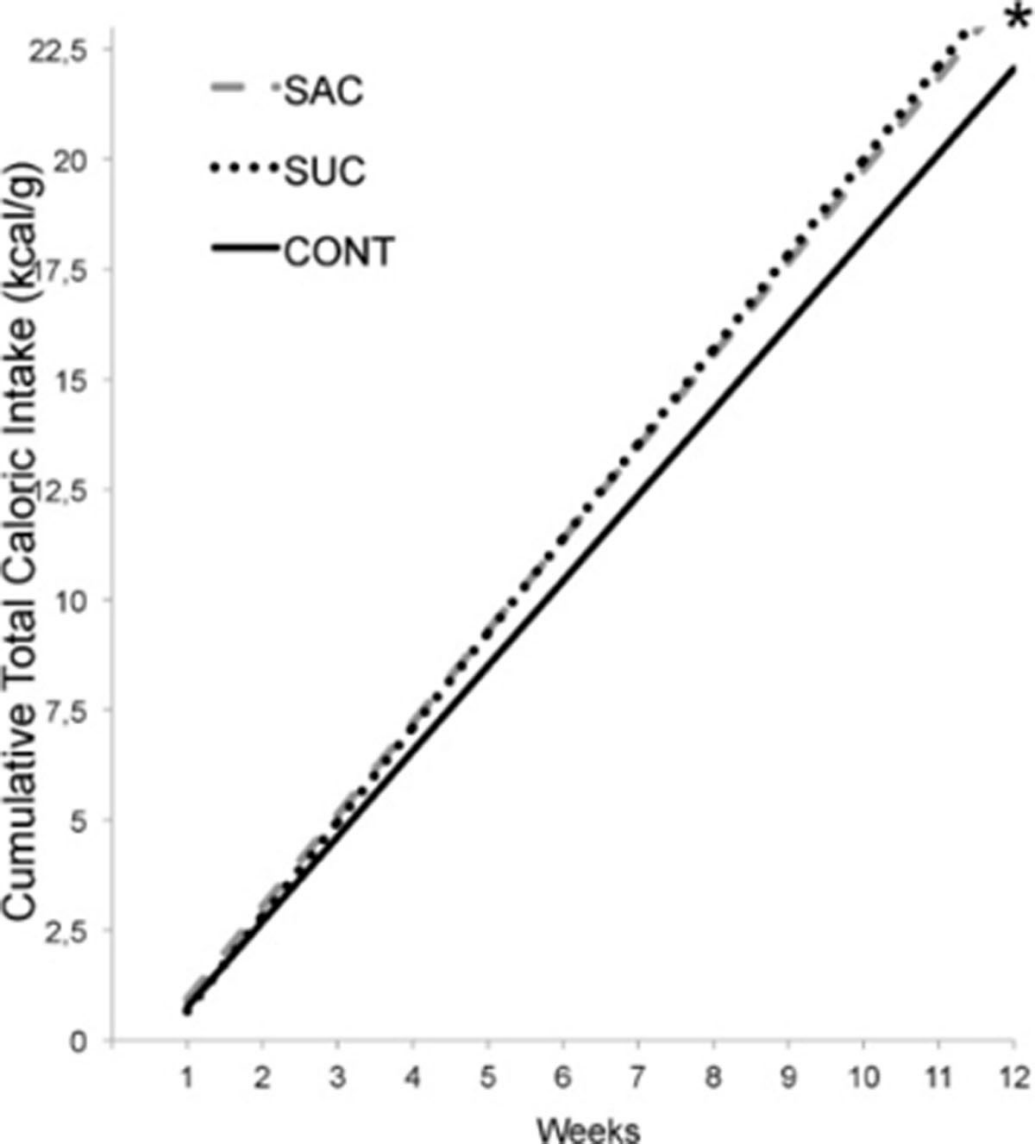
Cumulative total caloric intake connected by the weight weekly (kcal/g). Asterisks indicate comparisons in relations to SAC. * p <0.05 SAC in relations to CONT.

**Figure 3:**
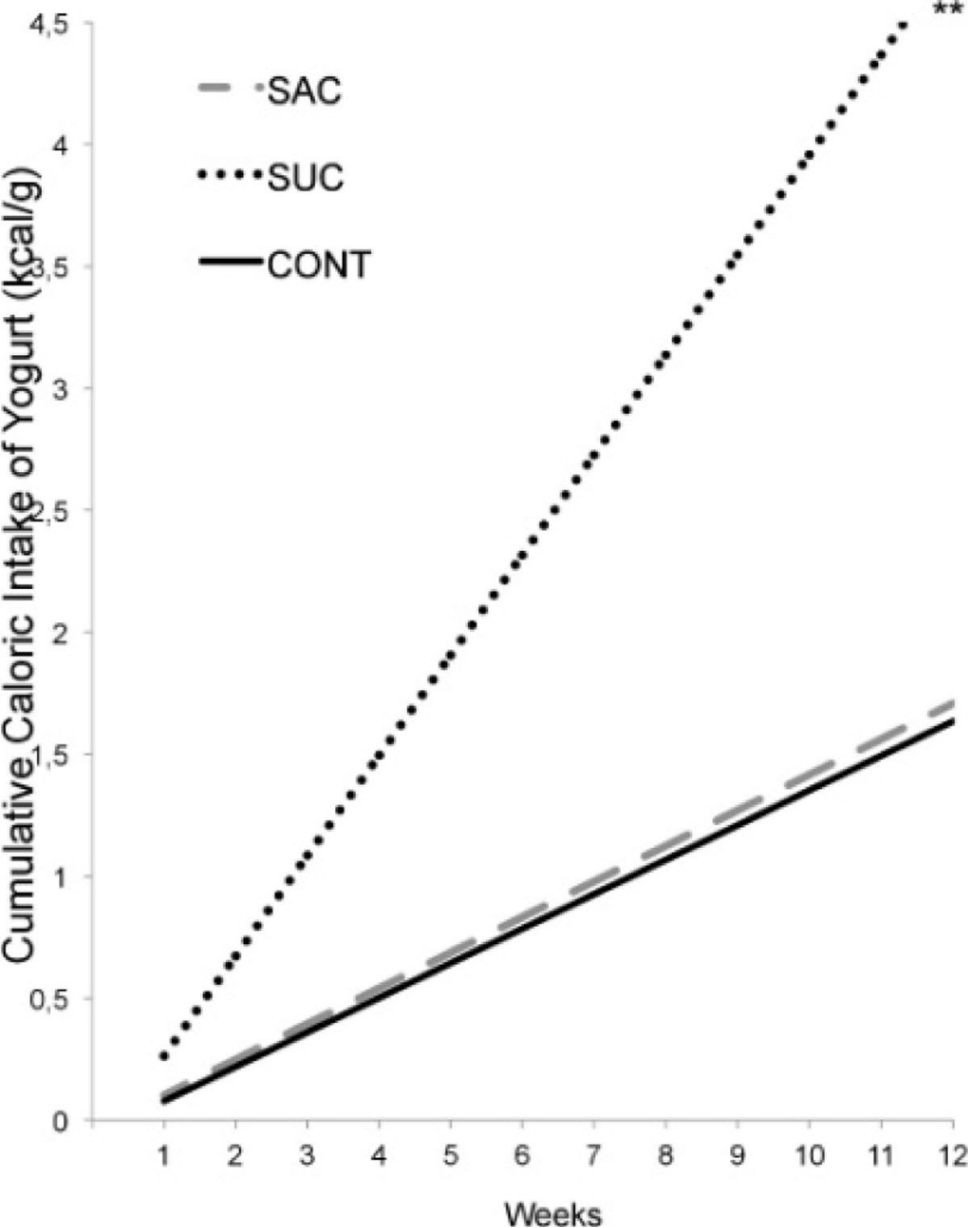
Cumulative caloric intake of yogurt connected by the weight weekly (kcal/g). Asterisks indicate comparisons in relation to SAC. ***, p < 0.001 SAC vs SUC.

**Figure 4:**
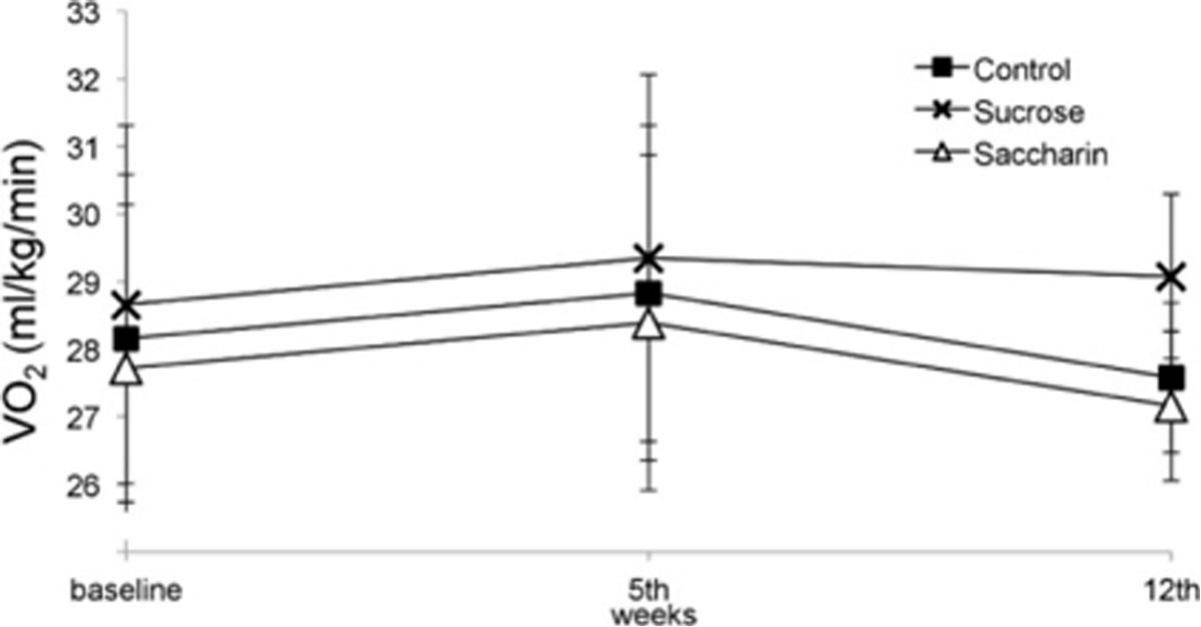
VO_2_ conducted on baseline, 5^th^ Wk and 12^th^ wk. Analysed by ANOVA of repeated measures.

**Figure 5:**
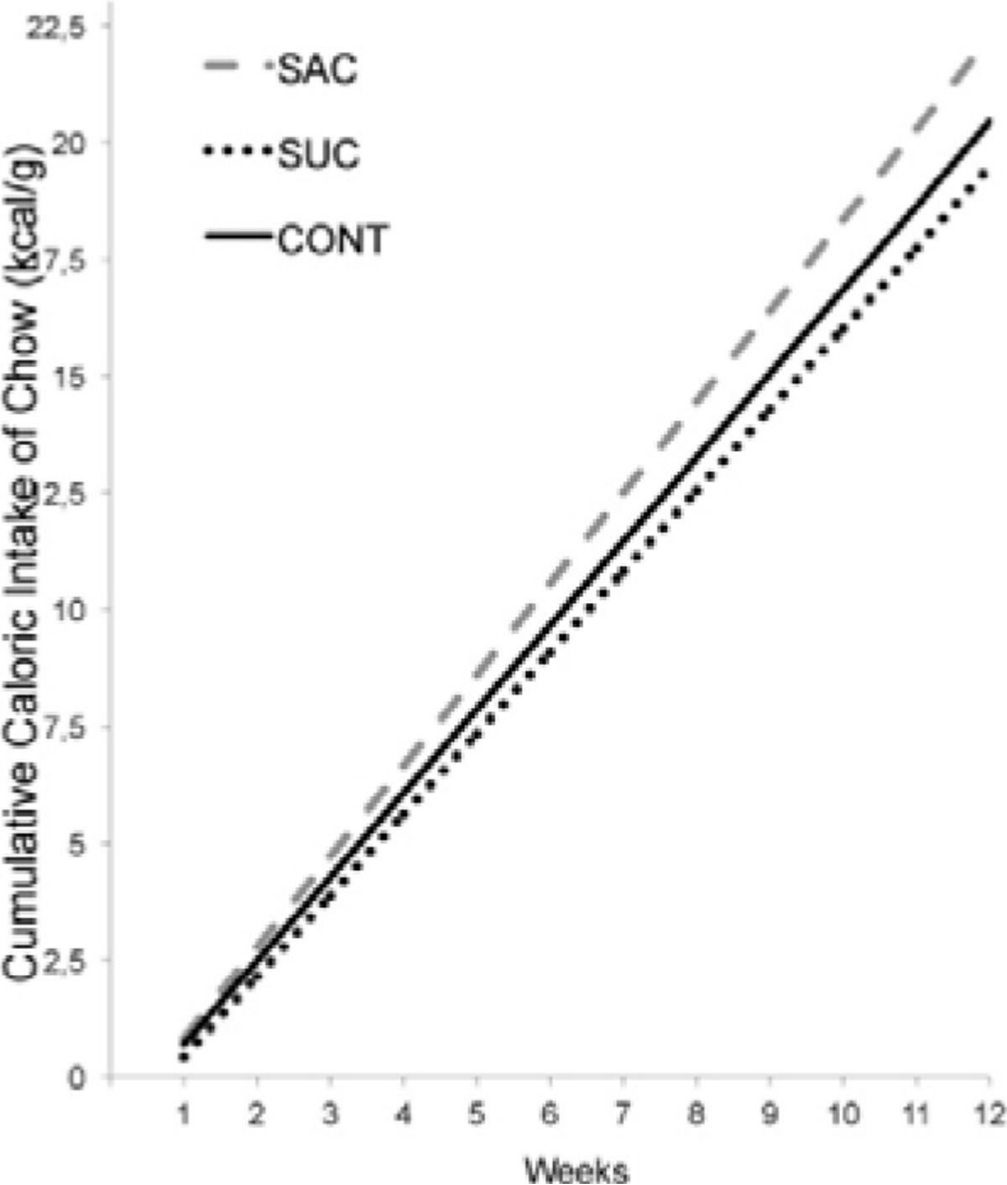
Cumulative caloric intake of chow connected by the weight weekly (kcal/g). Asterisks indicate comparisons in relation to SAC *p < 0.05.

**Figure 6:**
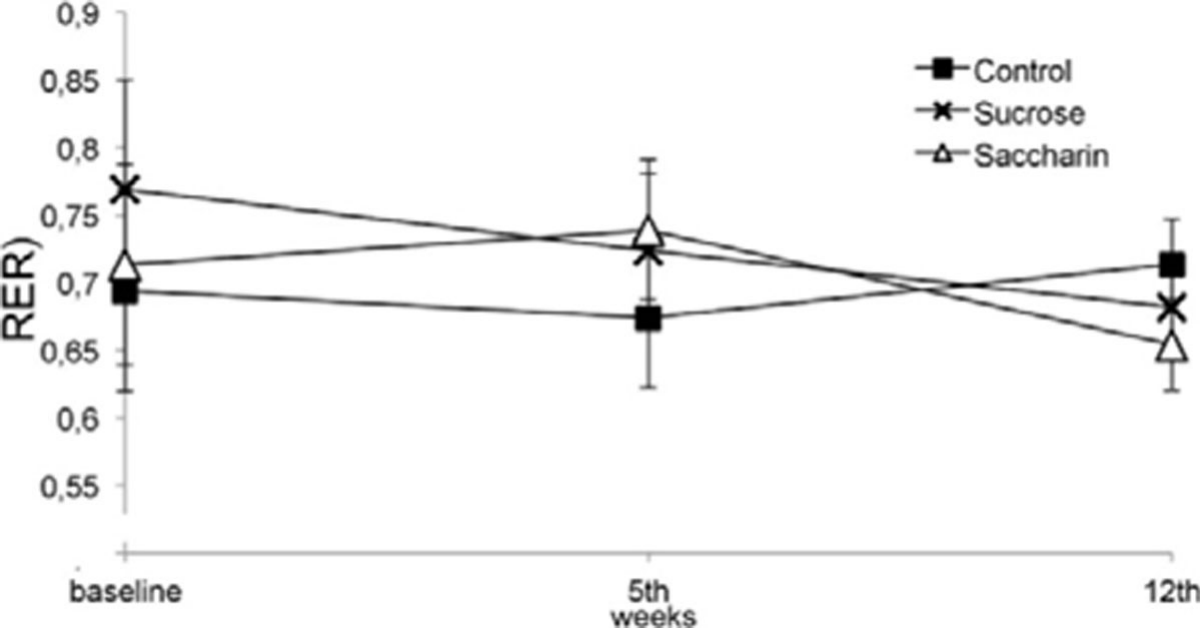
RER (respiratory exchange ratio) conducted on baseline, 5^th^ wk and 12^th^ wk. Analysed by ANOVA of repeated mesures.

**Figure 7:**
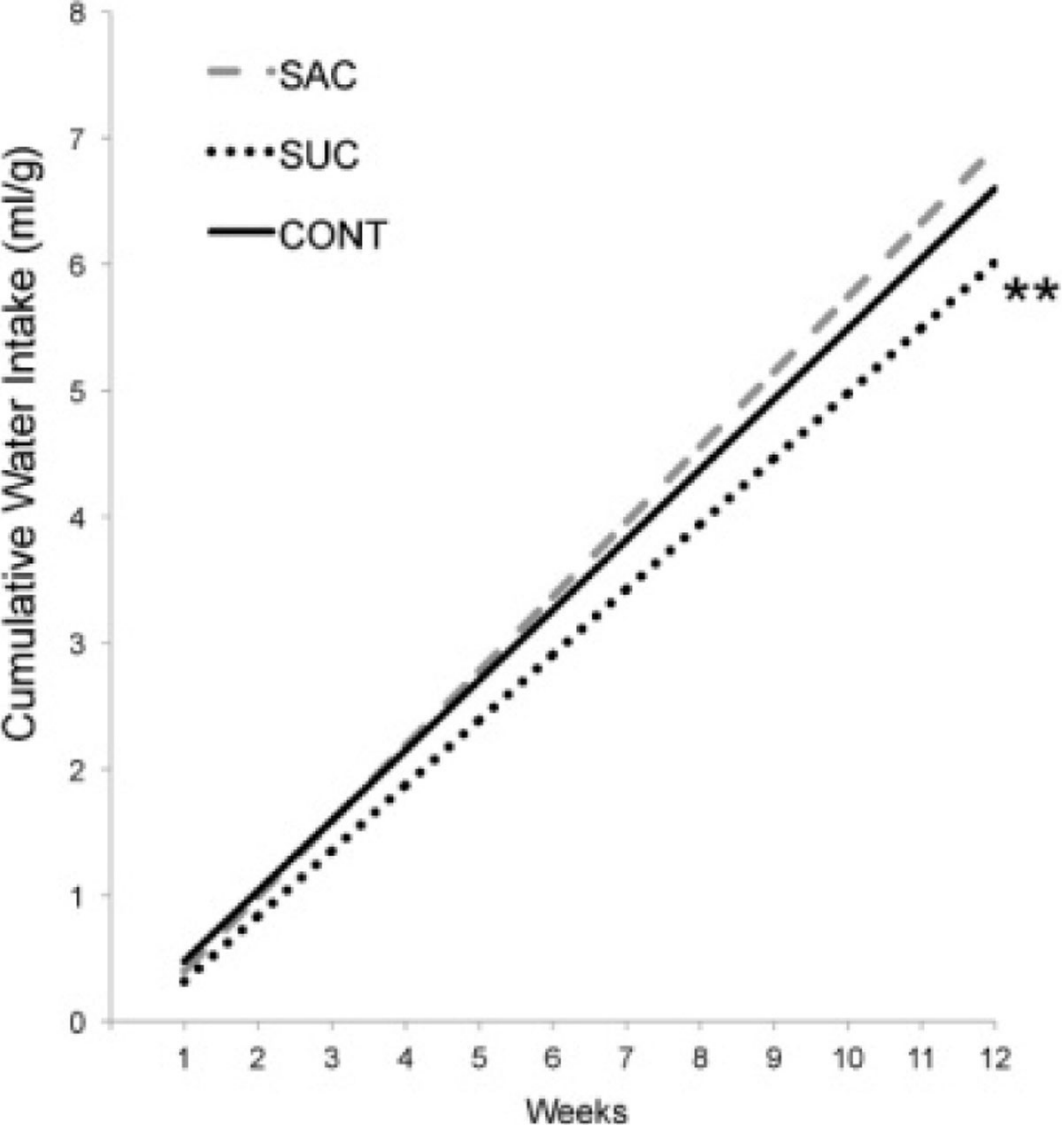
Cumulative water intake connected by the wright weekly (ml/g). Asterisks indicate comparisons in relation to SAC ** p < 0.005 SAC in relation to SUC.

**Figure 8:**
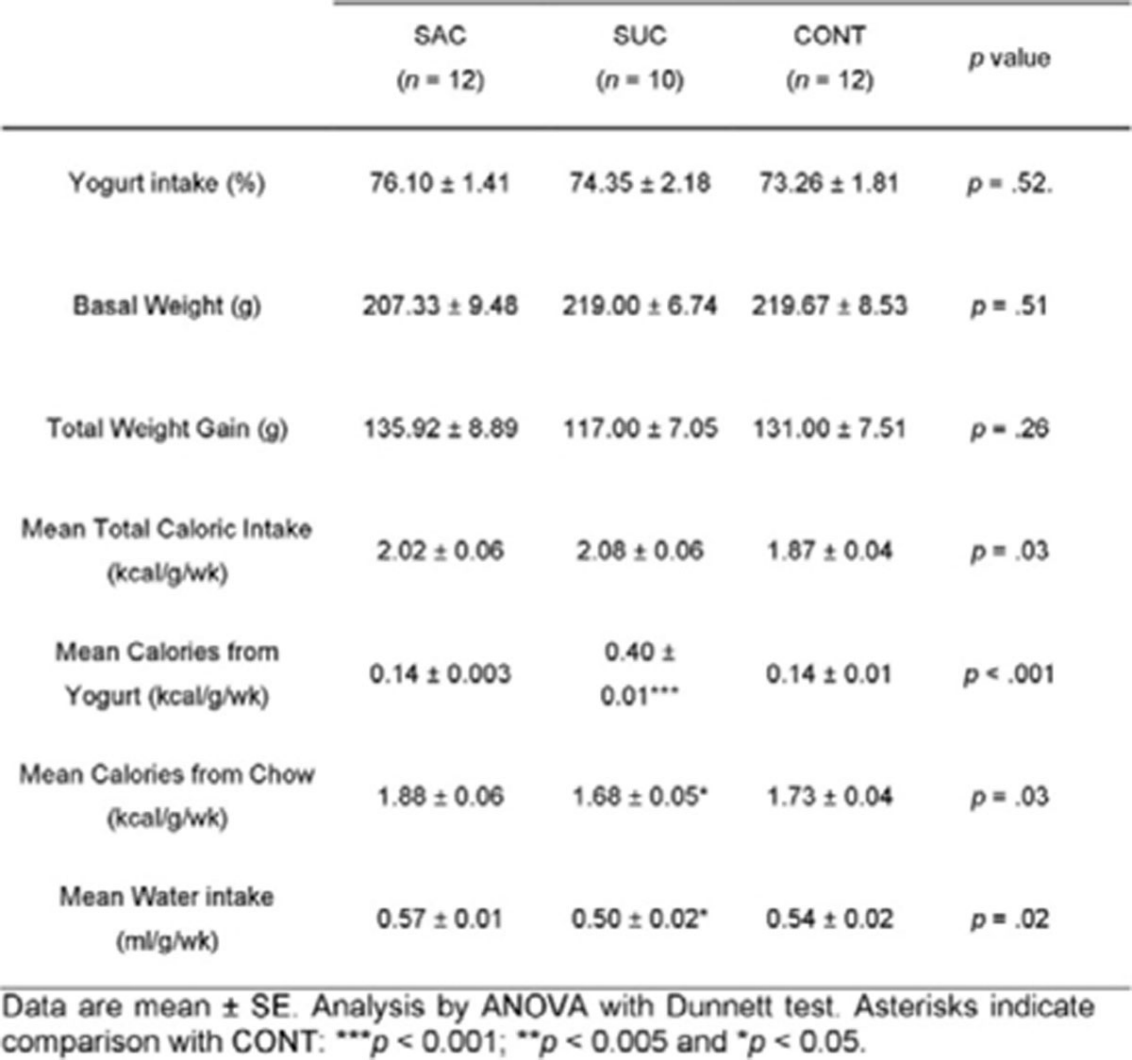
Weight, caloric and water intake parameters.

**Figure 9:**
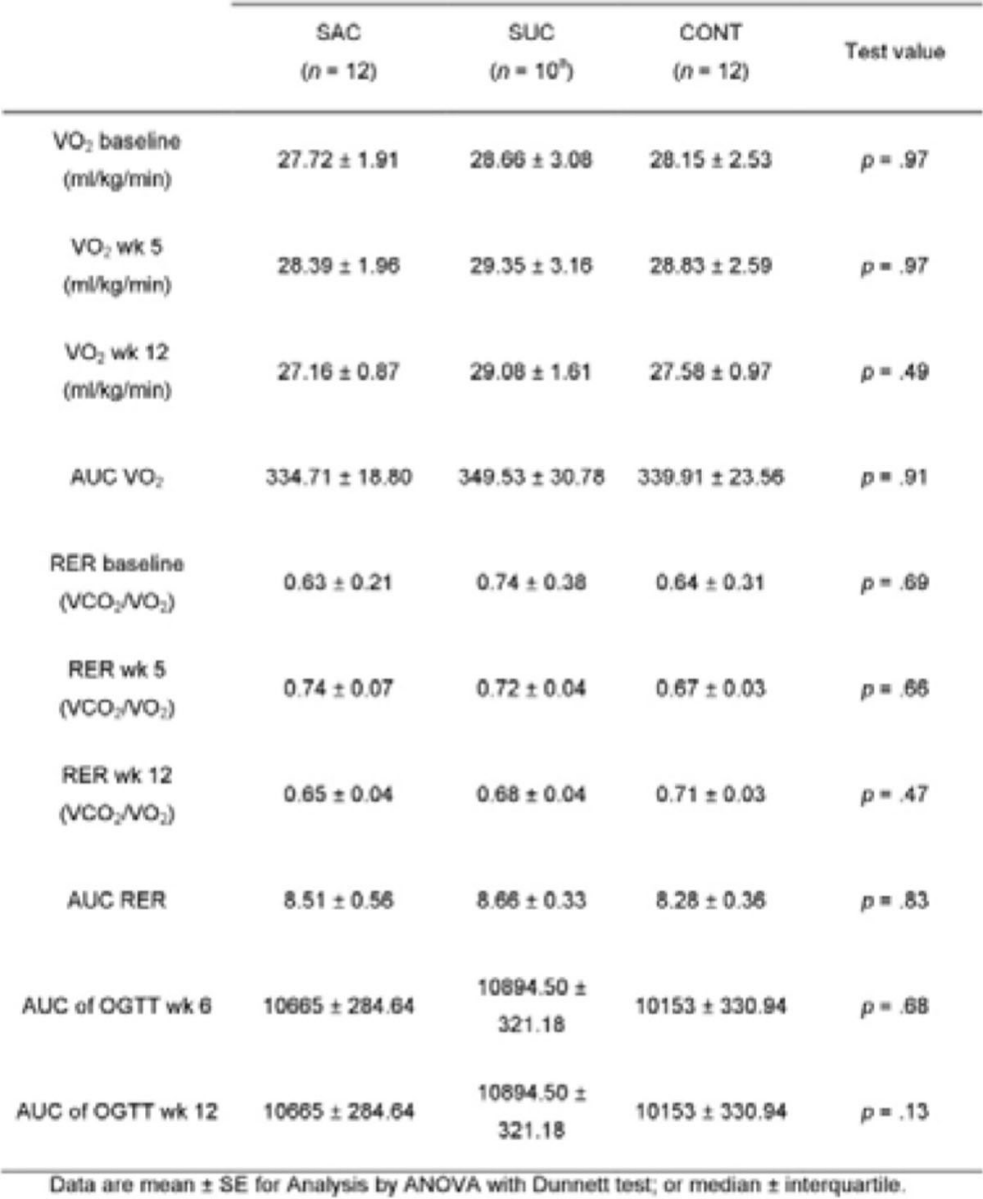
VO_2_, RER and OGTT parameters.

